# Wavelength, dose, skin type and skin model related radical formation in skin

**DOI:** 10.1007/s12551-021-00863-0

**Published:** 2021-11-26

**Authors:** M. C. Meinke, L. Busch, S. B. Lohan

**Affiliations:** 1 Center of Experimental and Applied Cutaneous Physiology, Department of Dermatology, Venerology and Allergology, Charité – Universitätsmedizin Berlin, Corporate Member of Freie Universität Berlin and Humboldt-Universität zu Berlin, Charitéplatz 1, 10117 Berlin, Germany; 2 Department of Pharmaceutics and Biopharmaceutics, Philipps-Universität Marburg, Robert-Koch-Str. 4, 35032 Marburg, Germany

**Keywords:** Electron paramagnetic resonance (EPR) spectroscopy, Reactive oxygen species, Lipid oxygen species, Sunscreen, UVC; Photodynamic therapy

## Abstract

The exposure to sun radiation is indispensable to our health; however, a long-term and high exposure could lead to cell damage, erythema, premature skin aging, and promotion of skin tumors. An underlying pathomechanism is the formation of free radicals which may induce oxidative stress at elevated concentrations. Different skin models, such as porcine-, murine-, human- ex vivo skin, reconstructed human skin (RHS) and human skin in vivo, were investigated during and after irradiation using X- and L-band EPR spectroscopy within different spectral regions (UVC to NIR). The amount of radical formation was quantified with the spin probe PCA and the radical types were measured ex vivo with the spin trap DMPO. The radiation dose influences the types of radicals formed in the skin. While reactive oxygen species (ROS) are always pronounced at low doses, there is an increase in lipid oxygen species (LOS) at high doses. Furthermore, the radical types arise independent from the irradiation wavelength, whereas the general amount of radical formation differs with the irradiation wavelength. Heat pre-stressed porcine skin already starts with higher LOS values. Thus, the radical type ratio might be an indicator of stress and the reversal of ROS/LOS constitutes the point where positive stress turns into negative stress.Compared to light skin types, darker types produce less radicals in the ultraviolet, similar amounts in the visible and higher ones in the infrared spectral region, rendering skin type-specific sun protection a necessity.

## Introduction

The skin is the primary interface between the body and the environment and is permanently exposed to physical, chemical, and biological environmental influences, such as solar radiation or pollution. Solar radiation is essential for human life as it is important for the stimulation of vitamin D synthesis and well-being. However, a too high dose of solar radiation also leads to the excessive formation of free radicals (Parrado et al. 2019). The amount of air pollutants is steadily increasing worldwide and causing major health problems; according to the WHO; more than 7 million people die each year from the combined effects of environmental and air pollution (Kuehn 2014). The pollution not only damages the lining of the lungs, but also affects skin health and has been linked to the development of skin diseases (Krutmann et al. 2014). All these external environmental influences, but also one’s own lifestyle and habits, such as cigarette and/or alcohol consumption or lack of sleep, can promote an increased generation of free radicals, enhancing the development of oxidative stress, which describes an imbalance between free radicals and the protective mechanisms of the metabolism (Aseervatham et al. 2013).

Free radicals are atoms or molecules that possess one or more unpaired free electrons and thus represent unstable, short-lived, and highly reactive molecules (Di Meo and Venditti 2020). They strive to compensate for their unstable state by wresting electrons from other molecules. The most important classes of free radicals in biological systems include reactive oxygen species (ROS), reactive nitrogen species (RNS), and reactive sulfur and carbon species (Weidinger and Kozlov 2015). Free radicals are regular products of cellular metabolism and are continuously generated in vivo (Valko et al. 2007; Wolfle et al. 2014). The primary ROS are very reactive and interact with lipids, DNA, and proteins in the microenvironment. As a result, secondary lipid radicals (LOS) and carbon-centered radicals (CCR) are formed (Zastrow et al. 2015; Albrecht et al. 2019a, b). Thereby, ROS need to be tightly controlled, in order to prevent their uncontrolled accumulation in the cell; otherwise, they are harmful to metabolic processes: an increased oxidation of cell components can be the consequence, enhancing cell and tissue damage. With regard to the skin physiology, ROS significantly contribute to the development of erythema, sunburn, inflammatory skin diseases, immunosuppression, skin cancer, and premature skin aging (Okayama 2005; Li et al. 2016).

### Measurements of radicals in skin

In general, the detection of metabolic radicals is difficult because of their low concentration and short lifetime. The electron paramagnetic resonance (EPR) spectroscopy enables the non-invasive quantification and characterization of free radicals in tissue (in vivo, ex vivo) and cell cultures. The following main events can be recorded by this method: (a) development of oxidative stress, (b) investigation of the antioxidative status, (c) penetration of active ingredients into the skin, and (d) structural analysis of molecules.

This review focuses on the effects of oxidative stress induced especially by irradiation with light of different wavelengths in different skin models and types as well as further applications, whereby the EPR technology is the main examination method. In all investigations, the quantification and/or characterization of radicals are the central topics.

**Fig. 1:**
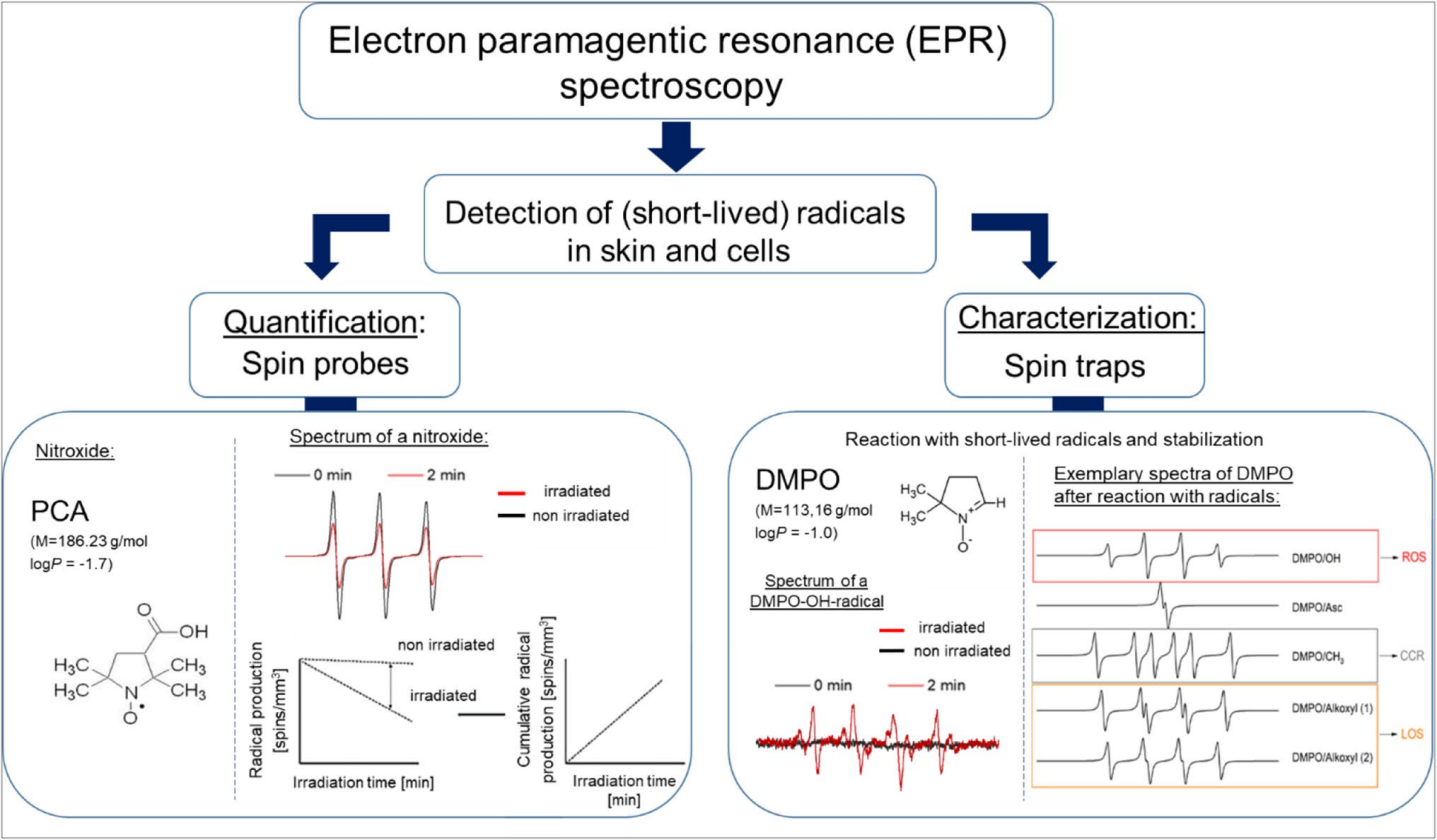
Schematic overview of the possibilities to investigate radicals in tissue and cells with the EPR spectroscopy. For the quantification of radicals, e.g., nitroxides can be used, the characterization can be carried out with the spin trap DMPO. Spin probes are often stable nitroxide radicals, whose EPR signal decreases with increasing radical concentration. After 2 min irradiation (red) of tissue treated with a nitroxide in comparison with a non-irradiated sample (0 min, black), a decrease in the spectrum intensity can be measured. The cumulative radical production can be calculated from the decrease over time and the difference to a non-irradiated sample (left side). The spin trap DMPO reacts with radicals and forms a more stable adduct. Initially, no EPR signal can be measured (0 min, black). Only after, e.g., irradiation which induces radical production, an EPR signal with DMPO can be detected (2 min, red), which can differ depending on the type of radical. The increase in the signal is proportional to the radical concentration. Exemplary spectra of the simulated radical species are shown: the hydroxyl radicals (DMPO/OH) defined as reactive oxygen species (ROS, red), the DMPO/CH_3_ abbreviated as C-centered radicals (CCR, gray), and the two alkoxyl species mentioned as lipid oxygen species (LOS, orange) (right side)

Most radicals are highly reactive and short-lived (Darr and Fridovich 1994); thus, they have to be marked or stabilized with specific substances, like spin probes or spin traps, in order to be detected in tissue (Jurkovic et al. 2003). Spin probes are especially used for radical quantification (Herrling et al. 2006; Albrecht et al. 2016); spin traps are used for the determination of radical types (Janzen 1984, Buettner and Mason 1990) (Fig. 1).

The use of, e.g., nitroxide spin probes allows statements about the formation of radicals (Haag et al. 2010; Lohan et al. 2016; Lohan et al. 2021a, b, c) or the endogenous redox state (Lauer et al. 2013; Lohan et al. 2020). In general, nitroxides are paramagnetic species with a free, unpaired electron in the outer shell of the oxygen atom. They have a characteristic EPR signal by themselves, which is why they are EPR-active. After reaction with a radical, they become EPR-inactive; thus, the intensity of the signal decreases with increasing radical production. The decrease in intensity of the spin probe PCA (3-(carboxyl)-2,2,5,5-tetramethyl-1-pyrrolidinyloxy) over the measured time enables the quantification of radicals in a biological system formed by exogenous factors, like irradiation (Albrecht et al. 2019a, b). The nitroxide 2,2,6,6-tetramethyl-1-piperidinyloxy (TEMPO) is a typical spin probe for the investigation of the antioxidative status (Lohan et al. 2015). Its amphiphilic character enables the uptake into cells, thereby the spin probe can directly interact with radicals produced by metabolic processes or with antioxidants (AOs). A decrease in the EPR signal of TEMPO over time enables conclusions about the redox status. In general, TEMPO can react with the antioxidant system in two directions, either it can be oxidized or it can be reduced but in skin the oxidation is mainly observed (Bacic et al. 2016). General statements about an oxidative imbalance can be made. In order to get a deeper insight into the change in the antioxidative status of cells, additional assay methods should be used that allow statements about endogenous antioxidants (Elpelt et al. 2019). In contrast to TEMPO, PCA is much more stable under experimental conditions, as shown in in vitro investigations with human keratinocytes (Walker et al. 2018).

With the EPR technology, semi-quantitative (Lohan et al. 2021a, b,c) but also quantitative measurements of free radicals in the skin (Albrecht et al. 2019a, b) can be made. Therefore, the quantitative EPR measurements have to be calibrated with an alanine standard with a defined spin number (2.01 × 10^17^spins) (Albrecht et al. 2019a, b; Lohan et al. 2021a, b,c). The accuracy of this quantification method is guaranteed by using a PCA solution with known concentration. It has to be taken into account that the concentration of the spin probe used and its applied amount significantly influences the detected cutaneous radical production as shown for PCA and N-tert-butyl-α-phenylnitrone (PBN). In parallel, it could be shown that PBN and PCA provide comparable results for the relative quantity and kinetics of radical production (Albrecht et al. 2016).

In addition to EPR spectroscopy, there are other detection methods available: from the determination of the total ROS concentration in tissue/cell culture to the specific detection of oxidation products of various cell components, e.g*.*, DNA (comet assay). These detection methods are primarily based on the detection of fluorogenic compounds by means of fluorescence spectroscopy or fluorescence microscopy (Di Meo and Venditti 2020). They are used frequently and corroborated by complementary techniques (Katerji et al. 2019). Nevertheless, light-scattering properties of the examined subjects may influence the evaluation, which can lead to misinterpretations and incorrect results. Furthermore, a number of reaction steps within the experiment can also lead to large errors (Kalyanaraman et al. 2012). The EPR spectroscopy provides more specific and sensitive results than fluorescence-based methods; using silver nanoparticles, it was demonstrated for the first time in vitro that the EPR technology is a more sensitive detection method for ROS production in cells compared to the fluorogenic dichlorofluorescein (DCF) assay (Ahlberg et al. 2016).

Spin traps can be used to determine the types of radicals. For example, the spin trap DMPO (5,5-dimethyl-1-pyrroline-N-oxide) can be used to differentiate between reactive oxygen species (ROS) and lipid oxygen species (LOS) in the skin (Albrecht et al. 2019a, b; Lohan et al. 2021a, b,c; Lohan et al. 2021a, b, c) (Fig. 1). In contrast to spin probes, no EPR signal is initially measurable. Only after the reaction with a radical, an EPR signal is detectable. In comparison to spin probes, spin traps have the advantage that their EPR spectrum enables the radical formed to be characterized by means of hyperfine coupling parameters. The proportions of radical species can be determined by simulating the spectra (Buettner 1987; Duling 1994; Marchand et al. 2017). The adducts formed, however, often only have a short half-life of seconds to a few minutes, depending on the spin trap, and are unstable and break down into further adducts (Bacic et al. 2008). In order to be able to detect the adducts of interest, optimized EPR settings with high sensitivity and an in situ stress application are required (Lohan et al. 2021a, b, c). Spin traps are currently only very rarely used in vivo, due to their instability in living tissue and their toxicity potential (Berliner et al. 2001; Khan et al. 2003).

### Irradiation induced radical formation

#### Quantification of radicals

The different spectral regions of sunlight (ultraviolet (UV), visible (VIS), and near infrared, NIR) play a major role concerning the amount of radical induction in skin. For Caucasian skin the highest radical formation is observed in the UVA spectral region (Zastrow et al. 2009). The radical formation beyond the UV is partly linked to enzymatic heat reaction (Schieke et al. 2003; Schroeder et al. 2008). The percentage of radical formation in the different wavelength regions depends on the skin type and skin model.

In vivo and ex vivo studies on human skin demonstrated that UV light generates most of the radicals, followed by VIS and NIR irradiation (Zastrow et al. 2009; Lohan et al. 2016). The radical concentration depends on the skin type (according to the Fitzpatrick scale (Fitzpatrick 1988)). Skin types I–III show a three times higher radical production after UV radiation than skin types IV–V: around 60% of all radicals are produced by UV, the remaining 40% of all free radicals are formed by VIS and NIR radiation (Lohan et al. 2016; Albrecht et al. 2019a, b). Compared to skin types I–III, skin types IV–V show a strongly reduced radical production in the UV, a comparable one in the VIS region, whereas in the NIR region the radical formation is higher. The reduction in the UV is related to the increased eumelanin content in skin of color compared to skin type II (Alaluf et al. 2002). Herrling et al. have shown ex vivo that the amount of melanin in the skin strongly diminishes UV-induced radicals up to 2.5 times (Herrling et al. 2008). The increase in the NIR is probably due to heat effects because of less light scattering. With increasing skin temperature, higher radical production was found in excised skin of type II after NIR irradiation (Zastrow et al. 2009). Heat and NIR irradiation independently multiply each other and lead to increased radical formation by interaction of the NIR with mitochondrion (COX activation leads to stimulation of mitochondrial ETC and ROS production) and absorption of radiation, which could increase the skin temperature and ROS production (Akhalaya et al. 2014).

**Fig. 2:**
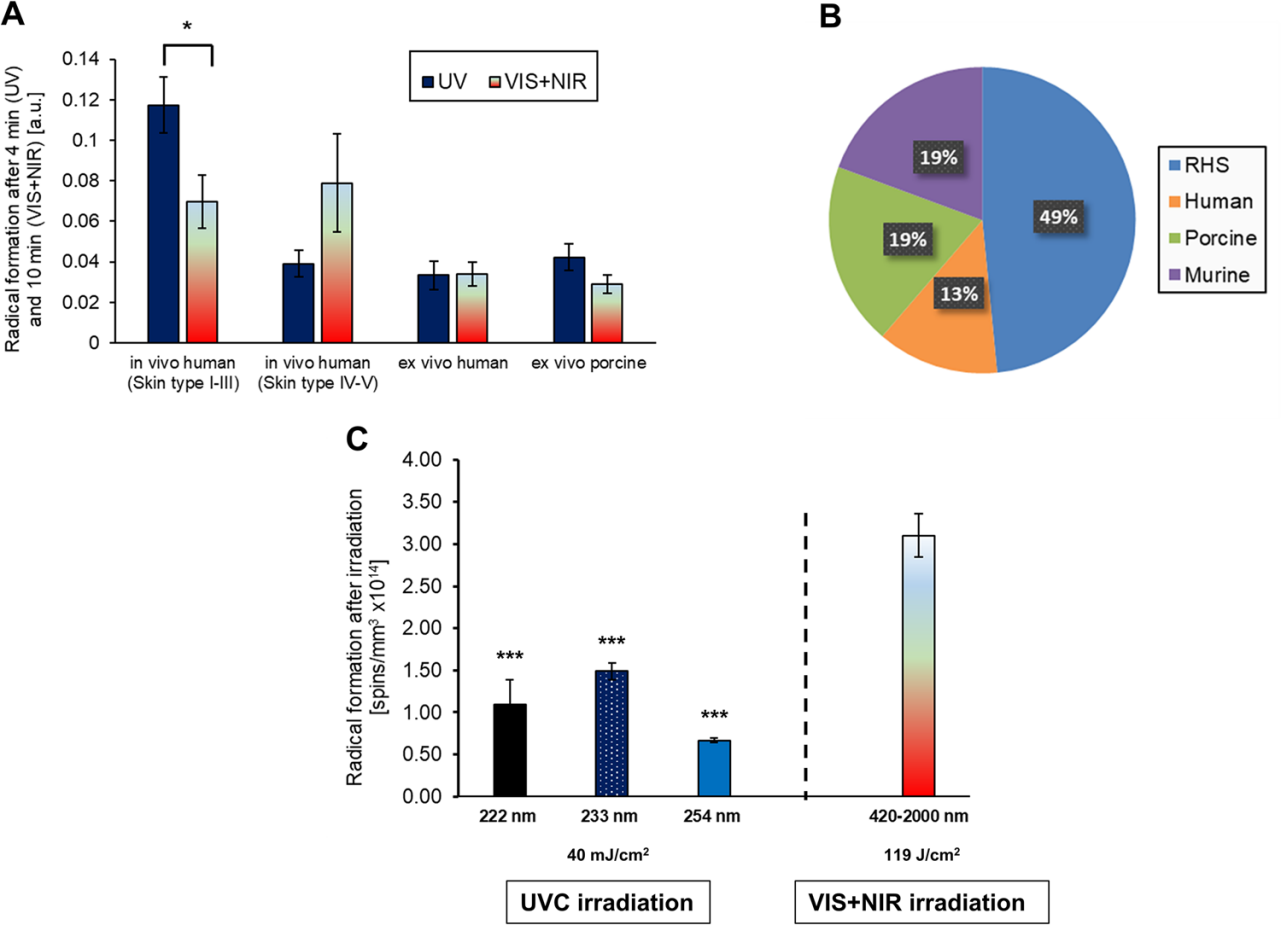
Radical formation in vivo (human), in vitro (reconstructed human skin, RHS) and ex vivo (human, porcine, murine) after irradiation with different spectral ranges of the solar spectrum. **A** Radical production in vivo and ex vivo measured with an L-band spectrometer for skin type II (*n* = 8 UV, *n* = 6 VIS and NIR) (Lohan et al. 2016), skin types IV-V (*n* = 6 UV, VIS and NIR) (Albrecht et al. 2019a, b) and for ex vivo skin (human skin, n = 6), porcine skin (*n* = 6)) during UV (302–375 nm, 1.6 mW/cm^2^, 4 min), VIS and NIR (420–2000 nm, 120 mW/cm^2^, 10 min) irradiation (Lohan et al. 2016); **B** comparison of the measured relative amount of radical production in % measured with an X-band spectrometer of different skin models (RHS and murine (*n* = 3), human and porcine (*n* = 4)) with the spin probe PCA (UVB-NIR irradiation (5.7 mW/cm^2^ (UVB-NIR), 0.028 mW/cm^2^ (UVB), 0.31 mW/cm^2^ (UVA)); **C** radical formation after irradiation of reconstructed human epidermal skin (RHS) models with UVC (222 nm (*n* = 5), 233 nm (*n* = 9) and 254 nm (*n* = 4)) at 40 mJ/cm^2^, VIS–NIR irradiation (420–2000 nm, 119 J/cm^2^, *n* = 3) served as control. Mean value ± s*tandard error* of *mean,* ^*^p ≤ 0.05,^***^p ≤ 0.001

Thus, for darker skin types, the radical load is reduced and more constant over the wavelength range. Nevertheless, dark skin types still produce 60% of the radicals of fair skin types. Sun protection should therefore be adapted to the skin type (Lan et al. 2018; Albrecht et al. 2019a, b). The combination of selected pigments, antioxidants, and chemical UV filters helps to ensure adequate protection in the entire spectral range. Together with coolants, a pleasant cooling effect on the skin for the entire spectral range is given (Lan et al. 2018). Excised fair human and porcine skin, these skin models generally show only one-third of the radical production as human skin does in vivo, for which the lack of oxygen in the excised tissue samples can be mentioned as the cause (Meinke et al. 2015) (Fig. 2A). Ex vivo skin or reconstructed skin models show also this limitation due to lack of perfusion which can be different in volunteer and patient skin (Fellmann 1992; Bollinger et al. 1996; Junger et al. 2000). Inflammatory skin diseases can also be replicated to a limited extent, only (Banerjee et al. 2021; Laclaverie et al. 2021). However, initial measurements on reconstructed skin models show differences in redox status between normal and inflamed skin (Elpelt et al. 2019).

**Fig. 3:**
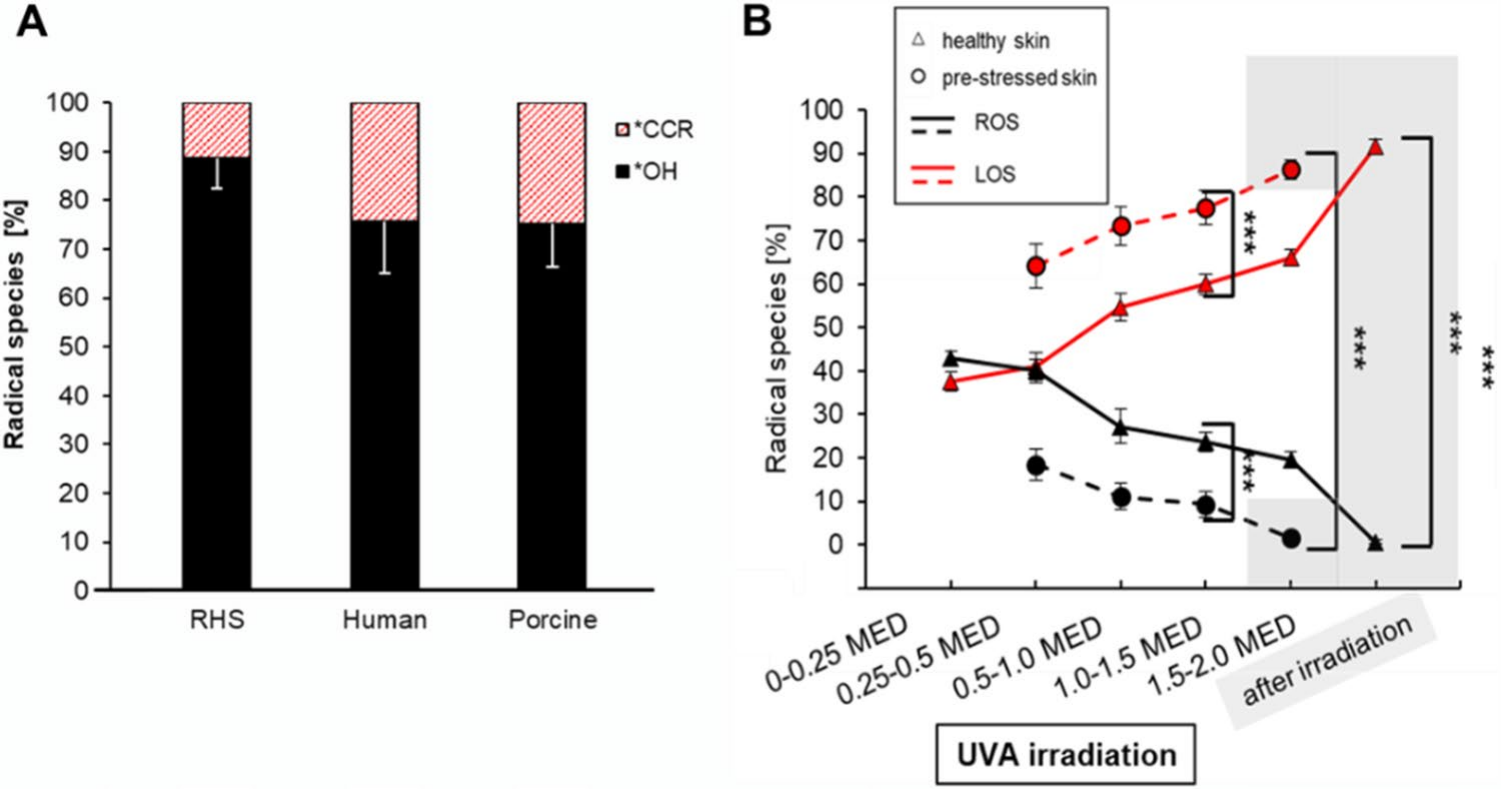
Characterization of radicals. **A** Relative radical concentration *OH/*CCR in % for RHS, human and porcine (*n* = 3 each), measured with DMPO (dose 0.53–1.0 MED (right) using an irradiance of 49 mW/cm^2^ (UVB-NIR), 0.24 mW/cm^2^ (UVB), 2.4 mW/cm^2^ (UVA)(Albrecht et al. 2019a, b); **B** percentage of radical species and progression of reactive oxygen (ROS) and lipid oxygen species (LOS) after irradiation of unstressed and stressed porcine skin (*n* = 10) with UVA (365 nm, 244 mW/cm^2^) (Lohan et al. 2021a, b,c). Mean value ± s*tandard error* of *mean,* ^***^p ≤ 0.001

For ex vivo skin models of different origins (human, porcine, murine), it could be shown that in vitro skin equivalents (reconstructed human skin, RHS) show the highest radical production after simulated solar irradiation (305–2200 nm, UVB-NIR) with a sun simulator. Since the RHS are supplied with nutrients up to the measurement vs. excised skin, they are metabolically more active, so that their radical production correlates better with the in vivo results than with those of the excised skin models (Fig. 2B); furthermore, more OH radicals are produced (Albrecht et al. 2019a, b) (Fig. 3A).

The different skin models contain different amounts of antioxidants (Thiele et al. 2001) which are important to neutralize free radicals (Pham-Huy et al. 2008). Porcine skin does not contain, e.g., carotenoids (Haag et al. 2010), which are important in light protection of the skin (Stahl and Sies 2012). Furthermore, human skin contains melanin which absorbs and scatters UV radiation (Prota 1980, Halliwell and Whiteman 2004) which is not available in the porcine skin used. Both differences could explain the enhanced radical formation in porcine compared to human excised skin. Murine skin shows strong variances in the structure to human and porcine skin; it is, e.g., much thinner (Gudjonsson et al. 2007). The intensity of sun irradiation decreases exponentially with the skin thickness (Bruls et al. 1984). The lower thickness could increase the radical formation in the deeper skin layers, because fibroblast are more sensitive to UV irradiation than keratinocytes (Otto et al. 1999). Considering the reduced thickness, a higher radical production in murine than in porcine skin would be expected; however, the amounts of radicals were comparable. Differences in the antioxidant defense system of murine and porcine skin could be the reason. Here, further investigations are necessary. All skin models produce the same radical species independent of type and origin. The fractions of oxygen-based or lipid oxygen-based radicals differ, but the influence of the irradiation dose is more pronounced. RHS exhibit more OH radicals than excised human and porcine skin due to the enhanced metabolism, which is closer to in vivo skin. Thus, porcine skin represents a good alternative as an excised skin model. Its morphology and functionality is very similar to human skin in contrast to murine skin (Jacobi et al. 2007), and is readily available as a waste product during slaughter, thus being a good alternative to in vitro models.

UVB irradiation induces mainly direct DNA damage (cyclobutane pyrimidine dimers (CPDs) and pyrimidine-(6–4)-pyrimidone photoproducts (6-4PPs) (Kielbassa et al. 1997). Radical production is here more downstream compared to UVA irradiation which causes high radical formation but low DNA damage (Zastrow et al. 2009). Already, 0.1 minimal erythema dose (MED) of UVB causes 80% DNA damage in the epidermal skin layer, and also some cell damage in the dermis can be observed (Zwicker et al. 2021).

UVC radiation represents the most energetic part of UV radiation. It does not hit the earth naturally, as it is almost completely absorbed in the atmosphere. Therefore, the effect on humans is so far scarcely known. Conventional UVC radiation with an emission wavelength of 250–280 nm is used in high doses to kill viruses and bacteria on surfaces, whereas it is not applied in humans due to its proven carcinogenic effect on skin and cornea. In contrast, particularly short-wave UVC radiation with wavelengths of 200–235 nm cannot penetrate the living cell layers of skin and wounds due to its high absorption in the first micrometers. Germ reduction as well as skin tolerance of the two wavelengths 222 nm and 233 nm were tested on germ carrier plates and skin models ex vivo/in vitro at different doses. Complete germ reduction takes place from 40 mJ/cm^2^ (Glaab et al. 2021). Based on these doses, irradiation studies were performed on intact excised human skin and reconstructed human skin (RHS) models. A 254-nm irradiation served as a positive control and visible light as a negative control. Radical formation at 40 mJ/cm^2^ for 222, 233 nm and 254 nm was well below 20 min of irradiation with VIS–NIR light. (Zwicker et al. 2021) (Fig. 2C).

### Characterization of radicals

The spin trap DMPO can be used to differentiate between ROS and LOS in skin. Zastrow et al. (Zastrow et al. 2015) have postulated that a universal threshold for free radicals in the body (free radical threshold value (FRTV)) is existing, which describes the *reversal* between beneficial and detrimental radicals and their consequential effects on the metabolism beyond the ground state. A significant dose dependence in radical production and a change of the ratio between ROS and LOS could be verified using different intensities of a UVA-LED irradiation unit (365 ± 5 nm) in ex vivo porcine skin. A reversal point after irradiation with the highest irradiance (~ 244 mW/cm^2^) could be detected in the range of 0.5 MED (Lohan et al. 2021a, b, c) (Fig. 3B) which for UVA is about 26 J/cm^2^ (Diffey and Farr 1989). Above 0.5 MED, LOS predominated over ROS. After switching off the irradiation, no further radical production could be quantified with PCA; with DMPO, changes in radical types were still observable after irradiation because of the stabilization effects of the trap, especially for LOS (Lohan et al. 2021a, b, c). High outside temperatures (> 35 °C) during animal husbandry and/or transport showed more LOS than ROS for the excised porcine ears at the beginning of the irradiation study, i.e., the reversal point had already been exceeded (Fig. 3B); nevertheless, the amount of radicals did not differ from that of unstressed skin. These results indicate that the skin was already severely stressed. In conclusion, it can be postulated that a certain amount of stress (activation stress) is necessary to detect a reversal in the ratio of ROS to LOS. This reversal indicates a change in the redox equilibrium. Low intensities of irradiance can still be compensated by adaptation processes of the skin. With the help of the newly established method, it is possible to characterize an already stressed system (LOS/ROS ratio shifts). The method requires in situ irradiation so that the radical quantification and characterization can be carried out without loss of time (Lohan et al. 2021a, b, c). In general, this reversal region (ROS to LOS) for radiation-induced radicals depends on the type of radiation (wavelength) and the dose.

### Further applications

*Sunscreen formulations* are known to protect against radicals generated by UV irradiation; however, only few data are known where such formulations also protect within the VIS/NIR spectral regions (Meinke et al. 2011; Souza et al. 2017). As shown, different skin types require their specific sun protection, particularly in the visible and near infrared spectral regions (Fig. 2A). For UV protection, two different types of UV filters are used: chemical and physical ones (Egambaram et al. 2020). Chemical filters convert the UV rays on the skin into heat, while physical filters (minerals, pigments) reflect the sunlight on the skin. Nevertheless, both filter systems are acting on the skin surface; thus, a protection of deeper skin layers is not achieved (Serpone et al. 2007). VIS and NIR irradiation penetrates deeper into the skin than UV light, enhancing an increased ROS formation in deeper skin layers (Meinke et al. 2011; Albrecht et al. 2019a, b). Due to the use of UV absorbing sunscreens, most consumers extend their exposure time in the sun by several hours without considering that they are not protected in the VIS/NIR spectral region.

Efficient sun protection, containing antioxidants (AOs) in addition to chemical and physical filters, as well as supplementation with AOs can also minimize the formation of radicals, and thus the development of associated skin diseases in deeper skin layers. In general, AOs are chemical compounds, which remove, detoxify, and/or slow down undesirable oxidation processes of cellular components, minimizing and/or impeding pro-oxidative effects (Berger et al. 2012). Endogenous AOs and those ingested with food (exogenous AOs) can be distinguished (Lu et al. 2010). For darker skin types, the antioxidants can be complemented by cooling substances to reduce the radical load in the infrared spectral region (Lan et al. 2018).

*The effect of AOs*, either implemented in cream or supplemented, can be investigated by EPR spectroscopy. Systemically administered AOs, if applied in physiological concentrations, can increase the AO status of the skin significantly, whereas water-soluble AOs penetrate more quickly into the skin, and reach a saturation concentration than lipophilic ones (Lohan et al. 2015). AO activity can significantly influence ROS formation and its consequences in the VIS and NIR spectral region, where typical sunscreen filters (chemical and physical ones) may no longer be effective (Yeager and Lim 2019). By EPR spectroscopy, it could be shown that an AO-containing sunscreen improved the photo-protection significantly in ex vivo skin with regard to VIS/NIR irradiation (Souza et al. 2017). Also in skin cells, a photo-protection was proven by AOs for this spectral region. In this study, it could be additionally demonstrated that a too high dose of beta-carotene has pro-oxidative effects (Lohan et al. 2018).

*Photodynamic therapy (PDT)* is a method for the treatment of tumors and skin diseases, in which a photosensitizer (PS) is irradiated with light of a certain wavelength, which causes generation of oxygen free radicals (photodynamic effect). These radicals promote, i.a*.*, the selective destruction of cancer cells (Kwiatkowski et al. 2018). For the first time, the production of radicals and the formation of radical types in general for a PS, here tetrahydroporphyrin tetratosylate (THPTS®), was investigated in porcine skin using EPR spectroscopy. An incubation time of 10 min promotes the highest radical production; a doubling of the penetration time does not lead to a double increased radical production in skin; the radical amount was reduced by a factor of 1.3. The longer penetration time is assumed to promote the probability of agglomeration of the photosensitizer molecules, whereby their functionality is impaired, resulting in a reduction of radical generation in the tissue.

## Conclusion

The highest radical formation due to solar irradiation occurs in the spectral regions UV (mainly UVA), but 40% of the produced radicals are induced by VIS and NIR. Depending on the skin model (in vivo, ex vivo, in vitro), different radical production regarding kinetics and quantity can be expected; reconstructed skin models represent the best model to in vivo skin due to the continuous supply of nutrients. Irrespective of the skin model, the irradiation dose per se influences the radical types formed in the skin. While ROS are always pronounced at low doses, an increase in LOS at high doses is given.

EPR spectroscopy enables the risk assessment of exogenous factors, such as the skin exposure to radiation of different spectral regions with regard to the induction of radicals. Topically applied cream formulations or the effect of supplementing antioxidants on skin health during UV, VIS, and NIR irradiation can be evaluated.
